# Risk factors and management of gastrointestinal bleeding in patients with or without antiplatelet and anticoagulation therapy: a multicenter real-world prospective study

**DOI:** 10.1186/s12876-024-03238-3

**Published:** 2024-05-07

**Authors:** Wenlin Hao, Anlei Liu, Huadong Zhu, Xuezhong Yu, Gang Chen, Jun Xu

**Affiliations:** 1 Emergency Department, The State Key Laboratory for Complex, Severe and Rare Diseases, Peking Union Medical College Hospital, Chinese Academy of Medical Science and Peking Union Medical College, Beijing, 100730 China; 2 Nephrology Department, The State Key Laboratory for Complex, Severe and Rare Diseases, Peking Union Medical College Hospital, Chinese Academy of Medical Science and Peking Union Medical College, Beijing, 100730 China

**Keywords:** Acute gastrointestinal bleeding, Antiplatelet therapy, Anticoagulation therapy, 28-day mortality, Blood transfusion

## Abstract

**Background:**

Antiplatelet and anticoagulation drugs complicate acute gastrointestinal bleeding (GIB) patients. Limited data about the risk factors and patient management has been presented. This study explored the association between previous antiplatelet or anticoagulant drug usage and clinical outcomes in GIB patients to improve awareness further and optimize treatment.

**Methods:**

We conducted a multicenter, non-interventional, real-world prospective study in 106 hospitals in 23 provinces in China. GIB patients confirmed in the emergency department were included and were grouped according to previous drug histories. Univariate analysis, multivariate logistic regression, and multivariate stratification models were performed separately to investigate the associations.

**Results:**

A total of 2299 patients (57.23 ± 17.21 years old, 68.3% male) were included, of whom 20.1% and 2.9% received antiplatelet and anticoagulation therapy, respectively. The all-cause 28-day mortality rates in patients without antiplatelet or anticoagulants, patients undergoing antiplatelet treatment, and patients with anticoagulation therapy were 2.8%, 4.6%, and 10.5%, respectively. After adjusting for confounding factors, both antiplatelet [odd ratio (OR), 2.92; 95% confidence interval (CI), 1.48–5.76; *p* = *0.002*] and anticoagulation therapy (OR, 8.87; 95% CI, 3.02–26.02; *p* < *0.001*) were associated with higher 28-day mortality. In the subgroup analysis, blood transfusion, especially red blood cell transfusion, in patients undergoing antiplatelet and anticoagulation therapy was associated with a decreased death risk.

**Conclusion:**

We confirmed an association between concurrent antiplatelet or anticoagulation therapy in GIB patients and elevated 28-day mortality. Blood transfusions could improve poor outcomes in such patients.

## Background

Gastrointestinal bleeding (GIB) is one of the leading issues in the emergency department (ED), with considerable morbidity and mortality. Previous studies reported that upper and lower GIB mortality rates ranged from 3.5% to 13% and 1% to 5%, respectively [1, 2].

Acute GIB represents the most frequent complication associated with antithrombotic medications [3–8]. With the increasing aging population worldwide and the usage of antiplatelets and anticoagulants for treatment and prophylaxis of cardiovascular diseases, the proportion of patients with GIB is expanding [7]. Extensive research has shown that antiplatelet and anticoagulation therapy significantly increase the risk of GIB [2, 9–13] and make treatment more challenging [7]. The main complication of antiplatelet and anticoagulation therapy is bleeding, with an annual risk of major bleeding of 2% to 4% [14]. In a population-based observational cohort study of patients who survived myocardial infarction, the rate of GIB was 1.5% per year with aspirin alone and 4.6% per year with aspirin plus clopidogrel or ticlopidine [15]. Additionally, among acute GIB patients, the prevalence of vitamin K antagonist usage is 8–15% for upper GI bleeding and 7% for lower GI bleeding [16]. Thus, awareness of these risks is critical to optimal management. So far, however, there hasn’t been much discussion on GIB mortality related to antithrombotic drug usage.

As part of the initial treatment of GIB, fluid resuscitation, transfusion, pharmacological measures, and correction of coagulopathy are administered to allow time for definitive surgical or endoscopic intervention directed at the site of bleeding [17]. However, individualized management plans should be developed based on bleeding etiology, comorbidities, and medication history in clinical practice to improve outcomes to the maximum extent [3, 7]. Currently, there is a lack of a complete and systematic clinical management process for selected GIB patients with a history of antiplatelet or anticoagulant medications.

Therefore, this study aims to discuss the relationship between previous antithrombotic therapy and GIB mortality in detail and to guide clinical treatment accordingly. We performed a multicenter, real-world prospective study to compare all-cause mortality from GIB in connection with antiplatelets and anticoagulants, as well as to analyze the main risk factors for increased GIB mortality in such patients to identify high-risk patients early and optimize the management and treatment strategies among GIB patients.

## Methods

### Description of definitions

The definition of GIB in this study was consistent with the latest guidelines of the American College of Gastroenterology (ACG). Upper gastrointestinal bleeding (UGIB) referred to bleeding originating from sites in the esophagus, stomach, or duodenum [18]. While lower gastrointestinal bleeding (LGIB) referred to hematochezia or bright red blood per rectum originating from a colorectal source [19]. All diseases addressed in the study were coded according to the ICD-10 system and all drugs mentioned as variables were harmonized according to the WHO-ATC classification codes.

### Patients selection

We conducted a multicenter, non-interventional, real-world prospective study to investigate GIB patients presented to the ED in 106 hospitals from 23 provinces in the People’s Republic of China between January 1st, 2015, and December 31st, 2016. The inclusion criteria for the study included: 1. adult patients with a confirmed GIB diagnosis based on the physician's judgment and the patient's chief complaints such as hematemesis, vomiting of coffee-like material, melena, and hematochezia; 2. patients or relatives who complied with the study and were able to provide written informed consent. We applied the exclusion criteria as follows: 1. exsanguinating hemorrhage caused by mechanical injury or other trauma; 2. pregnant or breastfeeding women; 3. patients were participating in other ongoing clinical studies. The Medical Ethics Committee has approved the study at Peking Union Medical College Hospital, which was the leading center of this study.

### Data collection and quality control

We consecutively enrolled patients during the study period and collected demographic characteristics, clinical data, laboratory examination results, and management methods from the medical records. Patient demographics included age, gender, province, and visiting hospital level. Clinical data included baseline blood pressure, vital signs in the ED, accompanying symptoms with GIB, current (within 1 week) antiplatelet or anticoagulation medicines before GIB events, history of combination usage of steroids and non-steroid anti-inflammatory drugs (NSAIDs, ATC:M01AH), and the cause of GIB. In our study, the antiplatelet drugs included aspirin (ATC: B01AC06), clopidogrel (ATC:B01AC04), tegretol (ATC:B01AC24), and dipyridamole (ATC:B01AC07), while anticoagulants included heparin (ATC:B01AB01), low molecular heparin (ATC:B01AB05), warfarin (ATC:B01AA03), and rivaroxaban (ATC:B01AF01). Steroids included prednisone (ATC:H02AB07), prednisolone (ATC:H02AB06), and methylprednisolone (ATC:H02AB04), while NSAIDs included acetaminophen (ATC:N02BE01), ibuprofen (ATC:M01AE01), and rosoprofen (ATC:M02AA31). Laboratory examination data included routine blood tests, urea nitrogen, and coagulation results during the ED visit. Management included medications for GIB treatment (including proton pump inhibitors, growth inhibitors, hemostatic agents and vasoactive drugs), blood transfusions, nasogastric tube placement, endoscope therapies, and surgical treatments. Proton pump inhibitors (PPIs) were commonly used in UGIB and included omeprazole (ATC:A02BC01), rabeprazole (ATC:A02BC04), and pantoprazole (ATC:A02BC02), while vasoactive drugs included norepinephrine (ATC:C01CA03), epinephrine (ATC:C01CA01), and posterior pituitary hormone (ATC:H01B). We investigated the 28-day mortality rate since ED admission as the primary outcome. Research assistants have contacted all patients enrolled or their relatives to ensure complete follow-up information. This was a non-interventional study, and the findings were primarily for descriptive purposes. No sample size calculations were involved in the study design.

In a formal kick-off meeting, we trained all coordinators and research assistants from all clinical research facilities. A key coordinator oversaw the project locally in each center. The sub-center coordinators and research assistants have collected patient data since ED admission. They also followed up on the enrolled GIB patients and continued data collection in endoscopy and operating rooms. The coordinators were responsible for reviewing and returning the complete form for each patient and transferring it into the central database monthly. There was a single location for checking the inherent logic and biological plausibility of patient data. We conducted all data queries within 30 days of raw data entry. To ensure the internal validity of the registry, we performed independent data validation on a random subset of all information collected by comparing 5% of all records with source data recorded in hospital charts quarterly. After assessing the prevalence and pattern of missing data, we found that it was not completely missing at random (quiz: *P* < *0.001*). Therefore, the study did not impute missing values.

### Statistical analysis

We conducted a descriptive analysis and compared the demographic characteristics, clinical data, laboratory examination results, management methods, and clinical outcomes among patients on antiplatelet drugs and anticoagulants and patients not on these drugs. Continuous variables were expressed as means ± standard deviation or medians with ranges, and categorical variables were expressed as frequencies and percentages. Continuous variables were compared using the t-test (for a normal distribution) or Mann–Whitney's U test (for a skewed distribution). Fisher's exact test was applied to compare the categorical variables. An univariate logistic regression analysis evaluated the risk factors associated with antiplatelet and anticoagulation therapy in patients with GIB. Non-adjusted and adjusted multiple logistic regression models were applied to assess GIB patients' antiplatelet and anticoagulant effects. The criteria for variable inclusion in the following regression analysis were based on both statistical significance in univariate analysis and clinical relevance. To ensure the clinical relevance of the selected variables, we conducted a comprehensive evaluation through collaborative discussions involving three experienced clinicians and one statistician. We performed the variance inflation factor (VIF) test for the variates in adjusted multiple logistic regression models to exclude the potential multicollinearity. Whether the covariances were adjusted was determined by the principle that the variables added to this model changed the matched odds ratio by at least 10%.

We performed all statistics with SAS, version 9.4 (SAS Institute Inc., Cary, NC, USA). The statistical significance was determined at *p* < 0.017 with 95% confidence intervals when we conducted multiple comparisons.

## Results

### Demographic description

**Fig.1:**
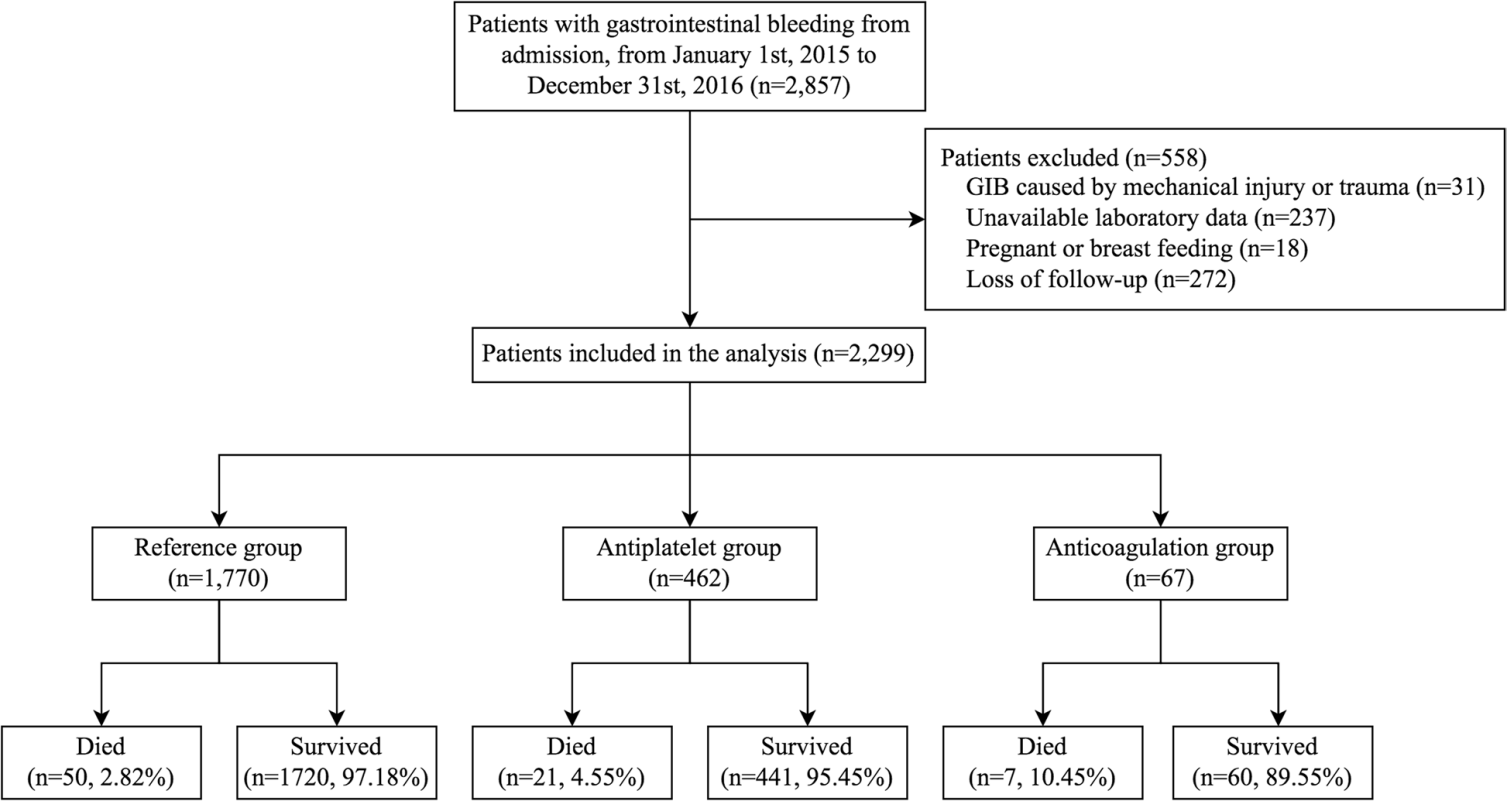
CONSORT diagram of patients. Shown is the flow of patients, illustrating treatment and control groups

After removing records with unavailable laboratory data and losing follow-ups, we included 2299 GIB patients from 106 hospitals in 23 provinces in this study. We divided patients into three groups according to current antiplatelet or anticoagulation medicines: patients with no antiplatelet drugs or anticoagulation used (the Reference group, 1770 cases), patients undergoing antiplatelet therapy (the Antiplatelet group, 462 cases), and patients with anticoagulation therapy (the Anticoagulation group, 67 cases). No patient was adjusted from antiplatelet to anticoagulant or from anticoagulant to antiplatelet in the week before GIB. There were no patients in our subgroup who took both antiplatelet and anticoagulant medications (Fig. 1).

The average age of patients enrolled was 57.23 ± 17.21 years, and 68.3% were male. We detected no difference in age, gender, or hospital level among all three groups. Melena was the most common complaint among all the patients (55.5%), followed by hematemesis (37.2%), vomiting of coffee-like material (17.1%), and hematochezia (15.3%). No significant differences were found in past GIB occurrences among these groups. Baseline hypertension occurred more often in the Antiplatelet group (81.4%) compared with the Anticoagulation group (44.4%) and the Reference group (33.3%) (*p* < *0.001*). We found that steroids (13.4%) and NSAIDs (11.9%) were most commonly used in the Anticoagulation group compared with the other two groups (*p* < *0.001* and* p* < *0.001*, respectively). Patients presented no difference in vital signs such as heart rate and mean blood pressure on ED admission. The average hemoglobin and platelet levels were 92.37 ± 28.40 and 180.33 ± 86.68 for all patients on admission. The hemoglobin and platelet tests showed no difference among the three groups, as did the international normalized ratio, prothrombin time, activated partial thromboplastin time, and urea nitrogen levels (Table 1).

**Table 1:** Baseline demographic and clinical characteristics

Characteristics	Total (*n* = 2,299)	Reference (*n* = 1,770)	Antiplatelet (*n* = 462)	Anticoagulation (*n* = 67)	*P*-value
**Age, mean ± SD**	57.23 ± 17.21	57.31 ± 17.27	56.99 ± 17.15	56.68 ± 16.33	0.91
**Gender, n (%)**					0.88
Male	1567 (68.3)	1204 (68.2)	319 (69.2)	44 (66.7)	
Female	726 (31.7)	562 (31.8)	142 (30.8)	22 (33.3)	
**Hospital level, n (%)**					0.20
Secondary	56 (2.5)	48 (2.7)	8 (1.8)	0 (0)	
Tertiary	2216 (97.5)	1703 (97.3)	446 (98.2)	67 (100)	
**Onset symptoms, n (%)**					
Hematemesis	856 (37.2)	653 (36.9)	168 (36.4)	35 (52.2)	0.035
Vomiting Coffee-like material	393 (17.1)	303 (17.1)	80 (17.3)	10 (14.9)	0.89
Hematochezia	351 (15.3)	286 (16.2)	57 (12.3)	8 (12)	0.094
Melena	1275 (55.5)	975 (55.1)	271 (58.7)	29 (43.3)	0.049
**Past GIB occurrence, n (%)**					0.98
First time	1561 (67.9)	1196 (67.6)	319 (69.1)	46 (68.7)	
Two times	85 (3.7)	66 (3.7)	17 (3.7)	2 (3)	
Multiple times	653 (28.4)	508 (28.7)	126 (27.3)	19 (28.4)	
**Past medical history, n (%)**					
Baseline hypertension	660 (44.2)	374 (33.3)	266 (81.4)	20 (44.4)	< 0.001*
Steroids use	52 (2.3)	27 (1.5)	16 (3.5)	9 (13.4)	< 0.001*
NSAIDs use	95 (4.1)	61 (3.5)	26 (5.6)	8 (11.9)	< 0.001*
**Vital signs on Admission, mean ± SD**					
Heart rate (bpm)	95.82 ± 18.18	95.67 ± 18.53	96.43 ± 17.08	95.64 ± 16.41	0.73
MBP (mmHg)	83.66 ± 15.91	83.83 ± 15.81	82.86 ± 16.65	84.73 ± 13.12	0.44
**Laboratory examination on Admission, mean ± SD**					
HGB (g/L)	92.37 ± 28.40	92.08 ± 28.76	93.52 ± 27.72	92.36 ± 22.95	0.63
PLT (× 10^9^/L)	180.33 ± 86.68	180.42 ± 87.12	182.46 ± 85.84	163.30 ± 79.56	0.25
INR	1.46 ± 1.99	1.44 ± 1.89	1.53 ± 2.33	1.47 ± 2.10	0.75
PT (sec)	14.69 ± 7.00	14.81 ± 7.27	14.20 ± 5.90	14.74 ± 6.48	0.28
APTT (sec)	32.51 ± 9.67	32.55 ± 9.85	32.18 ± 8.52	33.55 ± 11.79	0.53
Urea (mmol/L)	11.38 ± 9.01	11.29 ± 8.94	11.99 ± 9.67	9.58 ± 5.35	0.097

*Abbreviations*: *GIB* Gastrointestinal bleeding, *NSAIDs* Non-steroid anti-inflammatory drugs, *MBP* Mean blood pressure, *HGB* Hemoglobin, *PLT* Platelet, *INR* International normalized ratio, *PT* Prothrombin time, *APTT* Activated partial thromboplastin time

^*^ *P* < 0.017

### Treatment, diagnosis, and prognosis description

The treatment, diagnosis, and prognosis characteristics of GIB patients are listed in Table 2. Antiplatelet and anticoagulant therapy were discontinued in all patients after a definitive diagnosis of GIB. For non-invasive treatments, 97.13% of GIB patients were administered PPIs, followed by somatostatin (46.3%) and pituitrin (8.5%). Vasoactive drugs were used in 19.3% of all patients enrolled and 29% of the Anticoagulation group, with no significant difference. Blood transfusion was applied in 87.3% of the patients, among whom red blood cell transfusion was the most common (35.8%), followed by plasma (15%), platelets (2.5%), and cryoprecipitate (1.4%). We found no difference in the non-invasive treatments among all three groups. For invasive therapies, 20.7% of GIB patients received endoscope treatments, 4.5% received surgical therapies, and 2.4% received intervention therapies. Still, we found no difference in the invasive treatments among the different groups.

**Table 2:** Treatment, diagnosis, and prognosis characteristics

Medical processes	Total (*n* = 2,299)	Reference (*n* = 1,770)	Antiplatelet (*n* = 462)	Anticoagulation (*n* = 67)	*P*-value
**Treatment after admission, n (%)**					
Stomach tube input	395 (17.2)	309 (17.5)	79 (17.1)	7 (10.5)	0.33
CVC insert	444 (19.3)	339 (19.2)	89 (19.3)	16 (23.9)	0.63
PPI use	2233 (97.1)	1711 (96.7)	455 (98.5)	67 (100)	0.041
Somatostatin use	1065 (46.3)	798 (45.1)	227 (49.1)	40 (59.7)	0.025
Pituitrin use	196 (8.5)	158 (8.9)	29 (6.3)	9 (13.4)	0.066
Vasoactive drug use	390 (19.3)	294 (18.9)	78 (19.3)	18 (29)	0.14
Transfusion	2006 (87.3)	1553 (87.7)	395 (85.5)	58 (86.6)	0.43
RBC transfusion	823 (35.8)	636 (35.9)	161 (34.9)	26 (38.8)	0.80
Plasma transfusion	345 (15)	260 (14.7)	73 (15.8)	12 (17.9)	0.67
Platelet transfusion	57 (2.5)	42 (2.4)	12 (2.6)	3 (4.5)	0.54
Cryoprecipitate transfusion	32 (1.4)	20 (1.1)	12 (2.6)	0 (0)	0.035
Endoscope treatment	475 (20.7)	370 (20.9)	86 (18.6)	19 (28.4)	0.16
Surgery	104 (4.5)	82 (4.6)	16 (3.5)	6 (9)	0.12
Intervention therapy	55 (2.4)	41 (2.3)	10 (2.2)	4 (6)	0.15
**Diagnosis, n (%)**					
Gastric and duodenal ulcer	1221 (53.1)	906 (51.2)	275 (59.5)	40 (59.7)	0.003*
Esophageal and gastric varices	360 (15.7)	339 (19.2)	16 (3.5)	5 (7.5)	< 0.001*
GI Cancer	166 (7.2)	133 (7.5)	27 (5.8)	6 (9)	0.40
Gastritis and duodenitis	177 (7.7)	123 (7)	49 (10.6)	5 (7.5)	0.032
Mallory-Weiss syndrome	63 (2.7)	53 (3)	9 (2)	1 (1.5)	0.39
Esophageal ulcer	26 (1.1)	17 (1)	6 (1.3)	3 (4.5)	0.026
IBD	16 (0.7)	12 (0.7)	3 (0.7)	1 (1.5)	0.73
Dieulafoy syndrome	5 (0.2)	4 (0.2)	0 (0)	1 (1.5)	0.049
**Survival in 28 days, n (%)**					0.001*
Survived	2221 (96.6)	1720 (97.2)	441 (95.5)	60 (89.6)	
Died	78 (3.4)	50 (2.8)	21 (4.6)	7 (10.5)	

*Abbreviations*: *CVC* Central venous catheterization, *PPI* Proton pump inhibitor, *RBC* Red blood cell, *GI* Gastrointestinal, *IBD* Inflammatory bowel disease

*P < 0.017

Gastric and duodenal ulcers (ICD-10: K25, K26) (53.1%) still ranked as the most common diagnosis in GIB patients enrolled, followed by esophageal and gastric varices (ICD-10: I86.401) (15.7%), gastrointestinal cancer (ICD-10: C16-C18) (7.2%), gastritis and duodenitis (ICD-10: K29.901) (7.7%), Mallory-Weiss syndrome (ICD-10: K22.6) (2.7%), esophageal ulcer (ICD-10: K22.1) (1.1%), inflammatory bowel disease (ICD-10: K51.90) (0.7%), and Dieulafoy syndrome (ICD-10: K31.82) (0.2%). We found that more patients had gastric or duodenal ulcers in the Anticoagulation group (59.7%) compared to the Reference group (51.2%) and the Antiplatelet group (51.2%) (*p* = *0.003*). Otherwise, esophageal and gastric varices were most often seen in the Reference group (19.2%, *p* < *0.001*).

We calculated the 28-day mortality and found that the patients in the Anticoagulation group suffered from the worst prognosis, with a 10.5% death rate. This was significantly higher than that of the Reference group (2.8%) and the Antiplatelet group (4.6%) (*p* = *0.001*).

### Univariate analysis of risk factors associated with 28-day mortality

Using univariate analysis, we investigated the effect of risk factors associated with 28-day mortality in GIB patients (Table 3). There was a clear trend of increasing mortality in GIB patients with previous usage of anticoagulation therapy (OR 4.01 [95% CI 1.75–9.22], *p* = *0.001*). Other factors associated with increased 28-day mortality were being female (OR 1.70 [95% CI 1.08–2.68], *p* = *0.023*), baseline hypertension (OR 2.01 [95% CI 1.13–3.56], *p* = 0.017), using NSAIDs formerly (OR 2.39 [95% CI 1.07–5.35], *p* = 0.034), and having tachycardia on ED admission (OR 1.83 [95% CI 1.02–3.27], *p* = *0.042*). In contrast, higher hemoglobin levels were associated with decreased 28-day mortality. Compared to the group with a lower hemoglobin level, those with a hemoglobin level greater than 100 g/L on ED admission had a 50% reduction in 28-day mortality (OR 0.50 [95% CI 0.27–0.92], *p* = *0.026*). There was increased mortality in patients with the diagnosis of esophageal and gastric varices (OR 2.49 [95% CI 1.52–4.09], *p* = *0.0003*) or gastrointestinal cancer (OR 3.88 [95% CI 2.21–6.80], *p* < *0.001*), and a decrease in 28-day mortality when gastric or duodenal ulcers (OR 0.24 [95% CI 0.14–0.41], *p* < *0.001*) were diagnosed. There was no statistical significance for 28-day mortality in age, other vital signs, laboratory examination on admission, treatment after admission, or other diagnoses.

**Table 3:** Univariate analysis of risk factors associated with 28-day mortality

Risk factors	Statistics	OR (95%CI)	*P*-value
**Age (years), n (%)**			
Low (≤ 50)	752 (32.8)	1 [Reference]	
Middle (51–65)	770 (33.6)	1.33 (0.74, 2.39)	0.34
High (66–100)	768 (33.5)	1.54 (0.87, 2.73)	0.14
Age trend 1 year, mean ± SD	57.46 ± 14.67	1.01 (1.00, 1.03)	0.14
**Gender, n (%)**			
Male	1567 (68.3)	1 [Reference]	
Female	726 (31.7)	1.70 (1.08, 2.68)	0.023*
**Past medical history, n (%)**			
None	1770 (77)	1 [Reference]	
Antiplatelets use	462 (20.1)	1.64 (0.97, 2.76)	0.063
Anticoagulation use	67 (2.9)	4.01 (1.75, 9.22)	0.001*
Steroids use	52 (2.3)	1.77 (0.54, 5.82)	0.34
NSAIDs use	95 (4.1)	2.39 (1.07, 5.35)	0.034*
Baseline hypertension	660 (44.2)	2.01 (1.13, 3.56)	0.017*
**Vital signs on admission**			
Heart rate (bpm), n (%)			
Low (≤ 85)	722 (32.2)	1 [Reference]	
Middle (86–100)	759 (33.9)	1.39 (0.75, 2.55)	0.29
High (≥ 101)	761 (33.9)	1.83 (1.02, 3.27)	0.042*
Heart rate trend 1 bpm, mean ± SD	95.97 ± 14.65	1.02 (1.00, 1.03)	0.039*
MBP (mmHg), n (%)			
Low (≤ 76)	715 (31.9)	1 [Reference]	
Middle (77–89)	761 (34)	0.81 (0.47, 1.38)	0.43
High (≥ 90)	765 (34.1)	0.68 (0.39, 1.18)	0.17
MBP trend 1 mmHg, mean ± SD	83.31 ± 11.38	0.99 (0.97, 1.01)	0.17
**Laboratory examination on admission**			
HGB (g/L), n (%)			
Low (≤ 77)	743 (32.9)	1 [Reference]	
Middle (78–100)	759 (33.6)	0.98 (0.59, 1.63)	0.93
High (≥ 101)	754 (33.4)	0.50 (0.27, 0.92)	0.026*
HGB trend 1 g/L, mean ± SD	91.80 ± 23.26	0.99 (0.98, 1.00)	0.027*
PLT (× 10^9^/L), n (%)			
Low (≤ 135)	742 (33.2)	1 [Reference]	
Middle (136–207)	744 (33.3)	1.17 (0.67, 2.04)	0.58
High (≥ 208)	750 (33.5)	1.07 (0.61, 1.89)	0.80
PLT trend 1 × 10^9^/L, mean ± SD	176.95 ± 63.76	1.00 (1.00, 1.00)	0.82
INR, n (%)			
Low (≤ 1.05)	707 (32.5)	1 [Reference]	
Middle (1.06–1.2)	732 (33.6)	0.82 (0.46, 1.44)	0.48
High (≥ 1.21)	738 (33.9)	0.92 (0.53, 1.59)	0.76
INR trend 1.0, mean ± SD	1.19 ± 0.20	0.91 (0.29, 2.87)	0.88
PT (sec), n (%)			
Low (≤ 12.4)	714 (33.3)	1 [Reference]	
Middle (12.5–14.6)	708 (33)	1.38 (0.79, 2.38)	0.26
High (≥ 14.7)	725 (33.8)	0.90 (0.49, 1.63)	0.72
PT trend 1 s, mean ± SD	13.92 ± 2.23	0.97 (0.88, 1.08)	0.59
APTT (sec), n (%)			
Low (≤ 27.9)	697 (32.4)	1 [Reference]	
Middle (28–34.3)	735 (34.2)	0.63 (0.35, 1.15)	0.13
High (≥ 34.4)	718 (33.4)	0.97 (0.57, 1.65)	0.91
APTT trend 1 s, mean ± SD	31.54 ± 5.58	1.00 (0.96, 1.04)	0.94
**Treatment after admission, n (%)**			
PPI use	2233 (97.1)	1,539,905.04 (0.00, Inf)	0.98
Somatostatin use	1065 (46.3)	0.89 (0.57, 1.41)	0.62
Pituitrin use	196 (8.5)	1.42 (0.70, 2.89)	0.33
Transfusion	2006 (87.3)	0.80 (0.43, 1.49)	0.48
RBC transfusion	823 (35.8)	1.48 (0.94, 2.33)	0.091
Platelet transfusion	57 (2.5)	0.50 (0.07, 3.67)	0.50
Plasma transfusion	345 (15)	0.93 (0.49, 1.77)	0.82
Cryoprecipitate transfusion	32 (1.4)	0.00 (0.00, Inf)	0.98
Endoscope treatment	475 (20.7)	0.84 (0.46, 1.50)	0.55
Surgery	104 (4.5)	0.84 (0.26, 2.71)	0.77
Intervention therapy	55 (2.4)	0.52 (0.07, 3.82)	0.52
**Diagnosis, n (%)**			
Gastric and duodenal ulcer	1221 (53.1)	0.24 (0.14, 0.41)	< 0.0001*
Esophageal and gastric varices	360 (15.7)	2.49 (1.52, 4.09)	0.0003*
GI Cancer	166 (7.2)	3.88 (2.21, 6.80)	< 0.0001*
Gastritis and duodenitis	177 (7.7)	0.00 (0.00, Inf)	0.98
Mallory-Weiss syndrome	63 (2.7)	0.00 (0.00, Inf)	0.98
Esophageal ulcer	26 (1.1)	2.41 (0.56, 10.38)	0.24
IBD	16 (0.7)	0.00 (0.00, Inf)	0.98
Dieulafoy syndrome	5 (0.2)	0.00 (0.00, Inf)	0.99

*Abbreviations*: OR Odds ratio, *CI *Confidence interval, *NSAIDs* Nonsteroidal anti-inflammatory drugs, *MBP* Mean blood pressure, *HGB* Hemoglobin, *PLT* Platelet, *INR* International normalized ratio, *PT* Prothrombin time, *APTT* Activated partial thromboplastin time, *PPI* Proton pump inhibitor, *RBC* Red blood cell, *IBD* Inflammatory bowel disease

^*^ *P* < 0.05

### Multivariate logistic regression analysis of the association between the use of antiplatelet and anticoagulation therapy and 28-day mortality

We further analyzed the independent effects of antiplatelet or anticoagulation therapy on 28-day mortality in GIB patients. Multivariate logistic regression analysis was performed on the non-adjusted and two adjusted models (Table 4). In the non-adjusted model and adjusted model I (adjusted only for age and gender), the Anticoagulation group has a higher 28-day mortality than that of the Reference group (OR 4.01 [95% CI 1.75—9.22], *p* < *0.001* and OR 4.10 [95% CI 1.78—9.47], *p* < *0.001*, respectively). The adjusted model II was adjusted for age, gender, past medical history (baseline hypertension, steroids use, and NSAID use), vital signs on admission (heart rate, MBP), laboratory examination on admission (HGB, PLT, INR, PT, and APTT), and treatment after admission (PPI use, somatostatin use, pituitrin use, vasoactive drug use, transfusion, endoscope treatment, surgery, and intervention therapy). All the variates in adjusted model II were confirmed with a VIF value less than 5 to exclude the multicollinearity. In the adjusted model II, the 28-day mortality risk was 2.92 ([95% CI 1.48—5.76], *p* < *0.001*) times higher in the Antiplatelet group and 8.87 ([95% CI 3.02—26.02], *P* < *0.001*) times higher in the Anticoagulation group than that in the Reference group.

**Table 4:** Multivariate logistic regression analysis of the association between the use of antiplatelet and anticoagulation therapy and 28-day mortality

**Exposure**	**Non-adjusted** OR (95% CI), *P*-value	**Adjust I** OR (95% CI), *P*-value	**Adjust II** OR (95% CI), *P*-value
Antiplatelet vs. Reference	1.64 (0.97, 2.76) *P* = *0.063*	1.66 (0.98, 2.79) *P* = *0.058*	2.92 (1.48, 5.76) *P* = *0.002* *
Anticoagulation vs. Reference	4.01 (1.75, 9.22) *P* = 0.001*	4.10 (1.78, 9.47) *P* = 0.0009*	8.87 (3.02, 26.02) *P* < 0.0001*

Non-adjusted Model: adjusted for no risk factor

Adjusted Model I: adjusted for age and gender

Adjusted Model II: adjusted for age, gender, past medical history (baseline hypertension, steroids use and NSAIDs use), vital signs on admission (heart rate, MBP), laboratory examination on admission (HGB, PLT, INR, PT, and APTT) and treatment after admission (PPI use, somatostatin use, pituitrin use, vasoactive drug use, transfusion, endoscope treatment, surgery, and intervention therapy)

*Abbreviations*: *CI* Confidence interval

^*^P < *0.05*

### Multivariate stratification analysis of the association between the use of antiplatelet or anticoagulation therapy and 28-day mortality

We further conducted a multivariate stratification analysis of the association between antiplatelet or anticoagulation therapy usage and 28-day mortality in GIB patients (Table 5). The Antiplatelet and Anticoagulation groups were compared with the Reference group based on different stratification factors. As seen from the table, females could be a risk factor for GIB-related death by increasing the 28-day mortality in the Antiplatelet and Anticoagulation groups compared with the males, which was consistent with the results in the univariate analysis. Moreover, compared to patients with a lower heart rate, there was an increase in mortality in both groups when patients performed with a heart rate greater than 100 bpm on ED admission. The effect of blood transfusion on GIB-related death also needed to be emphasized, reducing the odds ratio of all-cause 28-day mortality from 3.38 (95% CI 0.82–13.90) to 1.47 (95% CI 0.84–2.60) in the Antiplatelet group and from 42.60 (95% CI 8.22–220.77) to 1.79 (95% CI 0.54–5.92) in the Anticoagulation group, respectively. This effect was most pronounced in the setting of red blood cell transfusions.

**Table 5:** Multivariate stratification analysis of the association between the use of antiplatelet or anticoagulation therapy and 28-day mortality

Stratification factors	All	Reference	Antiplatelet			Anticoagulation	
			**OR (95%CI)**		***P-value***	**OR (95%CI)**	***P-value***
**Gender**							
Male	1567	1	1.03 (0.49, 2.18)	0.94		1.69 (0.39, 7.28)	0.48
Female	726	1	2.96 (1.38, 6.35)	0.005*		9.43 (3.11, 28.55)	< 0.0001*
**Age (years)**							
Low (≤ 50)	752	1	1.29 (0.46, 3.64)	0.63		1.89 (0.24, 15.09)	0.55
Middle (51–65)	770	1	1.61 (0.62, 4.20)	0.33		10.83 (3.55, 32.98)	< 0.0001*
High (66–100)	768	1	1.92 (0.88, 4.19)	0.10		1.35 (0.17, 10.56)	0.77
**Past medical history**							
Baseline hypertension							
No	835	1	2.93 (0.81, 10.57)	0.10		10.78 (3.24, 35.86)	0.0001
Yes	660	1	2.25 (1.04, 4.89)	0.040*		5.82 (1.49, 22.83)	0.012*
Steroids use							
No	2247	1	1.62 (0.95, 2.76)	0.074		3.99 (1.64, 9.73)	0.002*
Yes	52	1	1.73 (0.10, 29.78)	0.70		3.25 (0.18, 58.06)	0.42
NSAIDs use							
No	2204	1	1.78 (1.04, 3.04)	0.036*		4.19 (1.71, 10.24)	0.002*
Yes	95	1	0.45 (0.05, 4.04)	0.47		1.60 (0.16, 15.74)	0.69
**Vital signs on admission**							
Heart rate (bpm)							
Low (≤ 85)	722	1	0.88 (0.25, 3.09)	0.84		2.32 (0.29, 18.67)	0.43
Middle (86–100)	759	1	1.28 (0.50, 3.28)	0.61		2.40 (0.53, 10.88)	0.26
High (≥ 101)	761	1	2.45 (1.16, 5.20)	0.020*		7.76 (2.36, 25.57)	0.0008*
MBP (mmHg)							
Low (≤ 76)	715	1	1.20 (0.50, 2.90)	0.68		5.19 (1.39, 19.38)	0.014*
Middle (77–89)	761	1	1.40 (0.55, 3.59)	0.49		2.78 (0.61, 12.69)	0.19
High (≥ 90)	765	1	2.68 (1.07, 6.67)	0.035*		4.59 (0.96, 21.81)	0.056
**Laboratory examination on admission**							
HGB (g/L)							
Low (≤ 77)	743	1	2.02 (0.90, 4.53)	0.086		1.96 (0.25, 15.60)	0.52
Middle (78–100)	759	1	1.62 (0.72, 3.66)	0.24		2.95 (0.82, 10.53)	0.096
High (≥ 100)	754	1	1.15 (0.31, 4.24)	0.83		10.74 (2.70, 42.81)	0.0008*
PLT (× 10^9^/L)							
Low (≤ 135)	742	1	1.99 (0.79, 5.01)	0.15		4.38 (1.19, 16.16)	0.027*
Middle (136–207)	744	1	2.75 (1.18, 6.43)	0.019*		12.84 (3.71, 44.49)	< 0.0001*
High (≥ 208)	750	1	0.81 (0.30, 2.19)	0.68		0.00 (0.00, Inf)	0.99
INR							
Low (≤ 1.05)	707	1	1.06 (0.39, 2.89)	0.90		2.98 (0.65, 13.72)	0.16
Middle (1.06–1.21)	732	1	2.21 (0.91, 5.38)	0.080		2.31 (0.29, 18.60)	0.43
High (≥ 1.22)	738	1	1.77 (0.71, 4.41)	0.22		6.32 (1.94, 20.55)	0.002*
PT (sec)							
Low (≤ 12.4)	714	1	1.17 (0.42, 3.24)	0.77		3.49 (0.75, 16.26)	0.11
Middle (12.5–14.6)	708	1	2.74 (1.26, 5.95)	0.011*		4.30 (0.91, 20.35)	0.066
High (≥ 14.6)	725	1	1.07 (0.35, 3.29)	0.91		5.00 (1.34, 18.63)	0.016*
APTT (sec)							
Low (≤ 27.9)	697	1	2.08 (0.93, 4.64)	0.074		1.41 (0.18, 11.14)	0.74
Middle (28–34.3)	735	1	1.33 (0.43, 4.15)	0.62		4.85 (1.02, 23.08)	0.048*
High (≥ 34.4)	718	1	1.29 (0.51, 3.29)	0.60		4.48 (1.22, 16.45)	0.024*
**Treatment after admission**							
PPI use							
No	66	1	1.00 (0.00, Inf)	1.00		NA	
Yes	2233	1	1.61 (0.96, 2.71)	0.074		3.88 (1.69, 8.90)	0.001*
Somatostatin use							
No	1234	1	1.81 (0.91, 3.62)	0.092		5.86 (1.90, 18.09)	0.002*
Yes	1065	1	1.46 (0.66, 3.21)	0.35		2.86 (0.82, 9.99)	0.010
Pituitrin use							
No	2103	1	1.55 (0.88, 2.70)	0.13		4.89 (2.10, 11.39)	0.0002*
Yes	196	1	2.92 (0.69, 12.42)	0.15		0.00 (0.00, Inf)	1.0
Transfusion							
No	293	1	3.38 (0.82, 13.90)	0.091		42.60(8.22, 220.77)	< 0.0001*
Yes	2006	1	1.47 (0.84, 2.60)	0.18		1.79 (0.54, 5.92)	0.34
RBC							
No	1476	1	1.84 (0.91, 3.71)	0.087		7.60 (2.93, 19.71)	< 0.0001*
Yes	823	1	1.45 (0.66, 3.16)	0.35		0.98 (0.13, 7.51)	0.98
Platelet							
No	2242	1	1.68 (1.00, 2.83)	0.052		4.21 (1.83, 9.70)	0.0007*
Yes	57	1	0.00 (0.00, Inf)	1.0		0.00 (0.00, Inf)	1.0
Plasma							
No	1954	1	1.56 (0.88, 2.76)	0.13		4.98 (2.13, 11.63)	0.0002*
Yes	345	1	2.10 (0.60, 7.36)	0.25		0.00 (0.00, Inf)	0.9937
Cryoprecipitate							
No	2267	1	1.66 (0.99, 2.80)	0.055		3.97 (1.73, 9.11)	0.001*
Yes	32	1	1.00 (0.00, Inf)	1.0		NA	
Endoscope therapy							
No	1824	1	1.40 (0.78, 2.52)	0.26		3.67 (1.38, 9.72)	0.009*
Yes	475	1	3.20 (0.99, 10.34)	0.052		6.10 (1.18, 31.61)	0.031*
Surgery							
No	2195	1	1.73 (1.02, 2.92)	0.042*		4.53 (1.96, 10.47)	0.0004*
Yes	104	1	0.00 (0.00, Inf)	1.0		0.00 (0.00, Inf)	1.0
Intervention therapy							
No	2244	1	1.67 (0.99, 2.82)	0.054		4.29 (1.86, 9.88)	0.0006*
Yes	55	1	0.00 (0.00, Inf)	1.0		0.00 (0.00, Inf)	1.0

*Abbreviations*: *OR* Odds ratio, *CI* Confidence interval, *NSAIDs* Nonsteroidal anti-inflammatory drugs, *MBP* Mean blood pressure, *HGB* Hemoglobin, *PLT* Platelet, *INR* International normalized ratio, *PT* Prothrombin time, *APTT* Activated partial thromboplastin time, *PPI* Proton pump inhibitor, *RBC* Red blood cell

*P < 0.05

## Discussion

This nationwide, multicenter study discussed the association between mortality from acute GIB and antithrombotic therapy. The sample size was relatively large, with more than 2,000 GIB patients presented to the emergency department included. We found that antiplatelets and anticoagulants were associated with an increased risk of all-cause 28-day mortality. Therefore, patients receiving antiplatelet or anticoagulation therapy should be considered a high-risk population for GIB, and early identification of these patients is an essential component of GIB management. For GIB in ED, a detailed history of prior medications should be obtained during the intake process. A scoring system may help identify patients with active gastrointestinal bleeding at an early time [20]. Another important finding was that transfusion therapy, especially red blood cell transfusions, may reduce the risk of death by 28 days. This may have significant implications for clinical practice and can guide improving the emergency department's medical care process with GIB. Improving the status of transfusion in managing GIB and optimizing the transfusion process may improve the prognosis of these patients.

In our study, 28-day mortality was highest in the Anticoagulation group, followed by the Antiplatelet group, and lowest in the Reference group. We performed multivariate logistic regression analysis, which suggested that anticoagulation or antiplatelet therapy was an independent risk factor for increased mortality with acute GIB. After adjusting for potential confounding factors, compared with patients without antithrombotic medications, anticoagulants increased the risk of 28-day mortality in GIB patients almost eightfold, while antiplatelet drugs increased the risk by about twofold. These results were consistent with those of Cuschieri et al*.,* who also found that patients using warfarin may be at an increased risk of GIB [20]. Yet the study only discussed GIB patients on warfarin and clopidogrel, whereas other antiplatelet drugs and various anticoagulants, including novel oral anticoagulants, were all included in our study. Furthermore, we also carried out more comprehensive statistical analysis methods, including univariate analysis, multivariate logistic regression analysis, and stratified analysis, all of which came to the same conclusion. Similarly, Tang and Sharma’s study found that GIB was significantly associated with warfarin and aspirin use [9]. However, the previous study only discussed anticoagulant and antiplatelet drug-related bleeding rates. At the same time, we took 28-day mortality after GIB as a primary outcome, which further illustrated the impact of antithrombotic therapy on the severity and poor clinical outcome of GIB. There were also similarities between the findings in this study and those described by Patel and Nigam, which showed that antiplatelet or anticoagulation therapy was associated with GIB mortality at 90 days and 6 months post-discharge [6]. However, they only included 716 patients with coronary heart disease. In contrast, our study had a larger sample size by including 2299 patients suffering from various comorbidities, and therefore, the validity of the conclusions was more substantial.

There are several different mechanisms by which antithrombotic agents may cause GIB. Antiplatelet drugs can increase the risk of gastrointestinal bleeding by causing ulcers and erosions at various levels of the digestive tract [21]. On the contrary, anticoagulants might precipitate bleeding from pre-existing lesions and interfere with the healing of the gastrointestinal mucosa [3]. Therefore, a certain population with gastrointestinal lesions such as peptic ulcers and gastrointestinal malignancies and patients with a history of bleeding are more likely to develop GIB when taking the drugs above, as many studies have already confirmed [2, 9, 22]. As a result, for these patients requiring antiplatelet or anticoagulation therapy, it is essential to establish a risk minimization strategy by carefully evaluating indications, minimizing the co-prescription of gastronomic drugs, and choosing the most appropriate dose of the most specific medicine according to the clinical characteristics of the patient [8]. Recent research has established that therapy with proton pump inhibitors (PPIs) can considerably lower GIB morbidity and mortality in antithrombotic therapy [16]. Combined PPIs may also be considered in patients at high risk of GIB [16], but further data validation is still needed.

Multivariate stratification analysis in this study observed a trend toward significantly decreased death risk in transfused patients, particularly those transfused with red blood cells, which could improve tissue oxygen delivery by boosting the blood’s oxygen-carrying capacity [17]. However, this finding contrasted with a previous retrospective cohort study enrolling nearly 60,000 GIB patients, which found a strong link between red blood cell transfusion and higher risks of hospital mortality and further bleeding [23]. Current international guidelines for GIB management recommend restrictive transfusion strategies (always defined indications for transfusion as hemoglobin less than 70 g/L) unless patients suffer shock or specific comorbidities [24]. This seems to align with earlier studies that discovered reduced mortality with restricted red blood cell transfusion as opposed to liberal transfusion [25–29]. However, these studies were all carried out in normal populations and might not apply to patients in co-therapy with antiplatelet drugs and anticoagulants. As discussed above, patients with antithrombotic therapy are at higher risk of GIB, so once the drug usage history is determined and the clinical diagnosis of GIB is established, transfusion of red blood cells should be considered to help improve the prognosis of these patients. Like the previous studies [17, 30], our results also failed to detect a significant statistical difference in the transfusion of other blood components, including platelets, plasma, and cryoprecipitate. This may suggest that targeted component transfusions are more meaningful for improving prognosis in patients with antiplatelet or anticoagulation therapy.

Additionally, this study had several limitations. First, the baseline data did not collect the dosage and duration of antiplatelet and anticoagulant drugs. As a result, a more detailed analysis of the degree of antiplatelet and anticoagulation therapy and dual or triple combination medications on GIB mortality was impossible. Second, the lack of a specific description of the underlying disease state may omit statistical consideration of risk factors for GIB. Third, the lack of baseline hemoglobin levels made it difficult to assess the proper volume of red blood cells to be transfused. Fourth, other unmeasured risk factors for GIB, including renal and hepatic insufficiency, elder age, and *H. pylori* infection [31–33] could skew the results. Thus, further clinical research is of great necessity to establish the safety and effectiveness of treatment approaches in GIB patients receiving antiplatelet and anticoagulation therapy.

## Conclusions

In this multicenter, prospective, non-interventional, real-world analysis of patients with GIB, antiplatelet, and anticoagulation medications were associated with higher rates of all-cause 28-day mortality. Transfusion therapy, especially red blood cell transfusion, improved clinical outcomes, and vigorous treatment strategies, including transfusion, were recommended for GIB patients receiving anticoagulation and antiplatelet therapy.

## Data Availability

The datasets used and/or analysed during the current study available from the corresponding author on reasonable request.
